# First-year growth patterns of preterm infants receiving kangaroo mother care: associations with early life factors and 1-year anthropometry

**DOI:** 10.1038/s41430-025-01662-6

**Published:** 2025-10-15

**Authors:** S. Nel, U. D. Feucht, T. Botha, M. Arashi, F. A. M. Wenhold

**Affiliations:** 1 University of Pretoria Faculty of Health Sciences, Department of Human Nutrition, Pretoria, South Africa; 2 University of Pretoria Research Centre for Maternal, Fetal, Newborn & Child Health Care Strategies, Atteridgeville, South Africa; 3 South African Medical Research Council (SA MRC) Maternal and Infant Health Care Strategies Unit, Atteridgeville, South Africa; 4 University of Pretoria Faculty of Health Sciences, Department of Paediatrics, Pretoria, South Africa; 5 Tshwane District Health Services, Gauteng Department of Health, Johannesburg, South Africa; 6 University of Pretoria Faculty of Natural and Agricultural Sciences, Department of Statistics, Pretoria, South Africa; 7 Deakin University Faculty of Health, Biostatistics Unit, Geelong, VIC Australia; 8 Ferdowsi University of Mashhad, Department of Statistics, Faculty of Mathematical Sciences, Mashhad, Iran

**Keywords:** Risk factors, Health care, Nutrition, Paediatrics

## Abstract

**Background:**

This study characterises first-year growth patterns in a historical preterm infant cohort, and investigates associated early-life factors and 1-year anthropometry.

**Methods:**

We analysed 322 South African preterm infants’ (mean 32.8 ± 2.4 weeks gestation) 1-year clinic records after kangaroo mother care discharge. Latent class trajectory modelling identified patterns of weight-for-age (WAZ), length-for-age (LAZ), weight-for-length (WLZ), and head circumference-for-age (HCZ) z-scores (Fenton 2013 Growth Chart; WHO Growth Standards, age-corrected). Z-score patterns were characterised as maintenance, faltering (progressively decreasing), gain (progressively increasing) or catch-up (rapidly increasing, exceeding birth z-score). Ordinal regression analysis investigated associations of early-life maternal/infant factors, birth weight, and early (until 50 weeks postmenstrual age) WAZ gain with growth patterns. One-year stunting (LAZ < -2), wasting (WLZ < -2) and overweight (body mass index-for-age z-score > +2) were compared.

**Results:**

Best-fit models identified three WAZ and LAZ patterns (gradual gain, faltering, catch-up), three WLZ patterns (maintenance, faltering, catch-up) and two HCZ patterns (maintenance, gain). Most infants displayed maintenance, gradual gain or catch-up. Lower birth weight z-score (BWZ) was associated with LAZ catch-up (OR:8.33 (3.13–20.00)), WLZ faltering (OR:2.94 (1.69–5.00)) HCZ gain (OR:1.92 (1.23–3.13)), but lower odds of gradual WAZ gain (OR:0.36 (0.19–0.68)) and WAZ faltering (OR:0.56 (0.34–0.92)). Smaller early WAZ gains were associated with gradual WAZ gain (OR:2.27 (1.56–3.33)), WAZ faltering (OR:1.47 (1.11,1.96)), LAZ catch-up (OR:1.85 (1.25–2.70)), and LAZ faltering (OR:1.39 (1.09–1.75)). WAZ and WLZ faltering were both associated (*p* < 0.001) with 1-year stunting (45.5%, 23.5%) and wasting (21.8%, 10.3%).

**Conclusions:**

Most preterm infants had appropriate first-year growth. Lower BWZ was associated with WAZ and LAZ catch-up but WLZ faltering, and sub-optimal early WAZ growth with growth faltering.

## Introduction

Infants born preterm (before 37 completed weeks’ gestation) and/ or small-for-gestational age (SGA) may exhibit different growth patterns than term-born, appropriate-for-gestational age (AGA) infants. In low-and-middle income countries (LMICs) [1–3], where nutritional and socioeconomic deprivation is common, these infants typically have incomplete catch-up growth and higher rates of childhood stunting, wasting and underweight than their high-income country (HIC) peers [4–7]. In LMICs and HICs alike, SGA preterm infants are more prone to persistent anthropometric deficits than those born AGA [2, 5–8].

Growth is best described as the change in an anthropometric parameter over time [9]. Most simplistically, it is difference in a measurement or z-score between two time points. This difference can also be expressed in terms of the baseline parameter (e.g. g/kg), time (e.g. g/day) or both (e.g. g/kg/day), though computational complexity increases with longer time periods [10]. In clinical practice, consecutive measurements are usually plotted on a sex-specific growth chart to visualise the infant’s growth curve. A healthy child’s growth curve is expected to run parallel to the chart’s reference curves, maintaining an approximately constant z-score over time – though wider intra-individual variations can sometimes be normal [9]. Additionally, neonates born after intrauterine growth restriction (frequently associated with preterm birth) typically display increasing z-scores (i.e. growth that is faster than the reference curve) as they return to their genetic growth potential with adequate nutritional intake. Reducing growth assessment to only the first and last measurement disregards potentially important information on intervening growth patterns.

Latent Class Trajectory Modelling (LCTM) techniques allow researchers to simplify and visualise longitudinal data by identifying characteristic trajectories within a group that share similar traits [11–13]. Thus, a large number of infants’ growth curves can be simplified to a few (usually 2–4) representative patterns, with each individual infant assigned to the pattern that best matches their individual growth curve [12–14]. LCTM has been used to investigate several growth outcomes, including length growth patterns and their relationship to stunting [15], relationships between linear and ponderal growth [16], childhood body mass index (BMI) trajectories and their associated determinants and outcomes [17], and foetal growth trajectories in relation to various outcomes [18, 19]. Application of LCTM methods to analysing the postnatal growth of preterm infants, however, remains under-researched.

This research primarily aimed to characterise the latent growth patterns of weight-for-age (WAZ), length-for-age (LAZ), weight-for-length (WLZ) and head circumference-for age (HCZ) z-scores during the first year of life in a cohort of South African preterm infants who received kangaroo mother care (KMC) during early life. Additionally, the relationships of these growth patterns to selected early-life factors (including maternal, pregnancy and neonatal conditions, size at birth and early growth according to weight z-score changes up to 50 weeks postmenstrual age (PMA)) and anthropometric outcomes (underweight, stunting, wasting and overweight) at 1 year were investigated.

## Methods

### Sample selection

This historical cohort study analysed existing infant records from the KMC post-discharge clinic at a tertiary academic hospital in a low-income peri-urban area of Tshwane District (Gauteng Province, South Africa). Records were systematically selected in reverse chronological order, starting from December 2018. Eligibility criteria included preterm birth ( < 37 weeks gestational age (GA)), a recorded birth weight and GA, and postnatal anthropometry up to 1 year. Infants with major anatomic or genetic abnormalities were excluded. Minor congenital heart conditions associated with preterm birth, such as patent ductus arteriosus (PDA) and patent foramen ovale (PFO) were not excluded, since they are prevalent in the target population and thus of considerable clinical relevance. Sampling deliberately included SGA infants above the population prevalence, to facilitate meaningful statistical analyses in this vulnerable sub-population: a priori power calculations called for 130 each of SGA and AGA infants. This necessitated including all SGA infants’ records from 2012 to 2018, whilst records from 2018 to 2016 provided ample AGA infants. Clinical policies did not change materially during this time, and all infants received comparable care.

### Data collection

For the historical data, paper-based records had been created by the clinic physician and dietitian. Birth data had been transcribed from birth records: birth weight had been measured in the maternity unit using electronic infant scales, and the GA had been confirmed by Ballard score if pregnancy dates were uncertain.

Follow-up anthropometry had been done by one hospital dietitian following standard protocols. Weight had been measured naked, to 0.01 kg, using electronic infant scales. Length had been measured to 0.1 cm using a portable measuring mat (with fixed headboard and moveable right-angled foot piece) placed on a hard, level tabletop. Head circumference had been measured to 0.1 cm using a flexible, non-elastic measuring tape. The dietitian had collected infant feeding information, and the paediatrician had performed and recorded medical examinations.

For this investigation, data were captured to Excel in duplicate, and discrepancies resolved using the original records. Maternal and infant sociodemographic and health information, birth data and follow-up anthropometry were recorded. Chronological, postmenstrual, and corrected ages (in days) were calculated for each visit. Chronological age was calculated automatically by subtraction of dates, and PMA as the sum of birth GA and chronological age. For corrected age (CA), the days of prematurity (280 days minus birth GA) were subtracted from chronological age.

Birth weight z-score (BWZ) and percentile were calculated using the Fenton 2013 Growth Chart calculator (https://ucalgary.ca/resource/preterm-growth-chart/calculators), and classified as SGA ( < 10^th^ percentile), AGA (10^th^-90^th^ percentile, inclusive) or LGA ( > 90^th^ percentile). Up to 50 weeks PMA, z-scores for weight, length, and head circumference were calculated using the Fenton 2013 Growth Chart. Early growth was quantified as the change in weight-for-PMA z-score from birth to the final measurement recorded before 50 weeks PMA. From 50 weeks PMA onward, the WHO Growth standards (WHO Anthro: http://www.who.int/childgrowth/software/en/) were used to calculate WAZ, LAZ, WLZ BMI-for-age z-score (BMIZ) and HCZ, using corrected age. At the final visit, infants were assessed for underweight (WAZ < -2), stunting (LAZ < -2), wasting (WLZ < -2), and overweight (BMIZ > + 2).

### Data analysis

All analyses were performed with R (version 4.1.2, 2020; R Foundation for Statistical Computing, Vienna, Austria). Growth curves per infant were plotted as z-scores (y-axis) against PMA (x-axis) and extreme values examined for errors. The analysis was delimited to PMA 200-650 days (28.5–92.9 weeks PMA, the latter equivalent to a CA of ~12.2 months), and to infants with three or more data points. For WAZ, LAZ and HCZ, z-scores from the Fenton 2013 Growth Chart (up to 50 weeks PMA) were combined with age-corrected z-scores from the WHO Growth Standards thereafter [20]. For WLZ, only the WHO Growth Standards were used, starting from 40 weeks PMA.

Latent trajectories were identified using Latent Class Growth Analysis (LCGA) and Growth Mixture Modelling (GMM) approaches, as described by Herle et al. [12]. For each analysis, models identifying two to four classes were considered. The LCGA models incorporated a fixed intercept and slope per class, while different GMM models incorporated either a random intercept and fixed slope or a random intercept and random slope per class, with and without non-linear link functions. Residual plots were visually inspected for model bias. Model fit was assessed as described by Lennon et al. [11], using relative entropy ( > 0.5 considered acceptable) and per-trajectory Average of maximum Posterior Probabilities of Assignment (APPA, <0.7 excluded), Odds of Correct Classification (OCC, <5.0 excluded), and group size (*n* < 10% excluded). The lowest Bayesian Information Criterion (BIC) identified the best-fitting model per anthropometric index, which was used for further investigations. Each infant was assigned to one growth pattern per model and the mean (with 95% CIs) trajectories of the actual data were plotted. The plotted growth curves were then characterised and assigned a label according to the growth pattern they most closely resembled: growth faltering (z-score decreasing over time), maintenance (z-score remaining more or less consistent), gain (z-score increasing over time, with the term “gradual gain” used when there was more than one increasing growth pattern in one indicator) or catch-up (rapid increase in z-score that ended up exceeding the birth z-score).

Univariate analysis investigated maternal and infant early-life exposures (maternal age, gravidity, parity, HIV infection, timing of antiretroviral therapy initiation, maternal health conditions during pregnancy, infant sex, birth GA, BWZ, SGA/ AGA status, infant congenital heart conditions, multiple gestation and early growth) as determinants of growth pattern group membership, and significantly associated exposure variables were incorporated in multiple ordinal regression analysis. Associations between growth pattern group membership and 1-year malnutrition (underweight, stunting, wasting and overweight) were investigated using frequency distributions and Chi-Squared or Fisher’s Exact tests.

### Ethical considerations

Approval to conduct the study was obtained from the University of Pretoria Faculty of Health Sciences Research Ethics Committee (Protocol 227-2021) and the hospital (KPTH 23/2021). All data were processed anonymously. All methods were performed in accordance with the relevant guidelines and regulations.

## Results

### Sample description

The study included 322 infants with 4-16 visits (median 9, IQR 8–10) each, providing 2925 data points for WAZ, 1929 for LAZ and HCZ, and 1583 for WLZ at ≥40 weeks PMA. Table 1 describes the sample infants and mothers (*N* = 302, excluding 20 duplicate records from twin mothers). Mean maternal age was 29.5 ± 6.6 years, with 6.3% aged ≤19 years and 26.0% aged ≥35 years. Of 63 (20.9%) HIV-infected mothers, 15.9% received no antenatal antiretroviral treatment. No infant contracted HIV. Mean birth GA was 32.8 ± 2.4 weeks and BWZ −0.77 ± 0.96, including 103 (32.0%) SGA, 216 (67.1%) AGA and 3 (0.9%) LGA infants. A comparison of the characteristics of SGA, AGA and LGA infants can be found in the Supplementary Table. Of 100 infants with non-critical congenital heart conditions, only one required surgical intervention. At 1 year, mean WAZ and LAZ remained <0, while WLZ and BMIZ were close to zero. At that age, the majority of the infants were within the z-score range of −2 to 2 for WAZ (87.0%), LAZ (82.0%), WLZ (81.7%), BMIZ (87.3%) and HCZ (89.2%). Rates of underweight (15.2%) and stunting (17.8%) were higher than wasting (6.9%) and overweight (6.5%). Associations of different growth patterns with these outcomes are described in the next section.

**Table 1:** Maternal and infant characteristics at birth and 1 year.

Characteristic	*n*	Value
**Maternal** ^**a**^		
Maternal age (years) [Mean ± SD]	285	29.5 ± 6.6
▪ Adolescent: age ≤19 years [*n* (%)]		18 (6.3)
▪ Advanced maternal age: ≥35 years [*n* (%)]		74 (26.0)
Gravidity (number of pregnancies) [median (IQR)]	285	2 (2; 3)
▪ Primigravida, Gravidity=1 [*n* (%)]		97 (34.0)
Parity (number of pregnancies carried to viable gestational age) [median (IQR)]	285	2 (1; 3)
▪ Primipara, Parity=1 [*n* (%)]		66 (23.2)
Maternal HIV infection [*n* (%)]	302	63 (20.9)
▪ Received ART during pregnancy	63	44 (69.8)
▪ No ART during pregnancy		10 (15.9)
▪ ART not recorded		9 (14.3)
Maternal conditions during pregnancy ^b^ [*n* (%)]	302	
▪ Conditions of the placenta, cord, membranes		13 (4.3)
▪ Pregnancy conditions		60 (19.9)
▪ Labor and delivery conditions		64 (21.2)
▪ Medical and surgical conditions		114 (37.7)
***Infant at birth***		
Infant sex (male) [*n* (%)]	322	160 (49.7)
Gestational age (weeks) [Mean ± SD]	322	32.8 ± 2.4
Birth weight (kg) [Mean ± SD] ^c^	322	1.65 ± 0.50
▪ Birth weight z-score ^d^ [Mean ± SD]		−0.77 ± 0.96
▪ SGA ^e^ [*n* (%)]		103 (32.0)
▪ AGA ^e^ [*n* (%)]		216 (67.1)
▪ LGA ^e^ [*n* (%)]		3 (0.9)
Infant is one of a set of twins [*n* (%)]	322	53 (16.5)
Infant congenital heart conditions ^f^ [*n* (%)]	322	100 (31.1)
***Infant at 1 year***		
Chronological age (months) [Mean ± SD]	322	12.55 ± 0.63
Corrected age (months) [Mean ± SD]	322	10.90 ± 0.75
Still breastfeeding at last visit [*n* (%)]	322	184 (57.1)
Change in WAZ from birth to ≤ 50 weeks PMA^d^ [Mean ± SD]	320	−0.10 ± 1.14
Weight (kg) [Mean ± SD]	322	8.58 ± 1.43
▪ Weight-for-age z-score ^g^ [Mean ± SD]	322	−0.56 ± 1.36
▪ Weight-for-length z-score ^g^ [Mean ± SD]	321	−0.13 ± 1.31
▪ BMI-for-age z-score ^g^ [Mean ± SD]	321	−0.07 ± 1.29
Length (cm) [Mean ± SD]	321	71.35 ± 3.01
▪ Length-for-age z-score ^g^ [Mean ± SD]	321	−0.89 ± 1.17
Head circumference (cm) [Mean ± SD]	322	45.40 ± 1.77
▪ HC-for-age z-score ^g^ [Mean ± SD]	322	0.22 ± 1.28
Indicators of malnutrition [*n* (%)]		
▪ Underweight: Weight-for-age z-score ^g^ <-2	322	49 (15.2)
▪ Stunted: Length-for-age z-score ^g^ <-2	321	57 (17.8)
▪ Wasted: Weight-for-length z-score ^g^ <-2	321	22 (6.9)
▪ Overweight: BMI-for-age z-score ^g^ >+2	321	21 (6.5)

*ART* antiretroviral therapy, *BMI* body mass index, *HC* head circumference, *HIV* human immunodeficiency virus.

^a^ 20 duplicate records of mothers of 40 twins were removed: thus *N* = 302 mothers.

^b^ Maternal conditions classified according to WHO ICD10-PM categories [41]. Conditions of labour and delivery only include conditions other than preterm delivery, as preterm birth was an inclusion criterion for the study.

^c^ No reliable birth length or HC measurements were available.

^d^ Calculated using the Fenton 2013 Growth Chart [20]

^e^ SGA = small-for-gestational age: birth weight <10^th^ percentile; AGA = appropriate for gestational age: birth weight 10^th^-90^th^ percentile; LGA = large-for-gestational age: birth weight >90^th^ percentile. SGA infants were deliberately over-sampled for statistical analysis purposes; the overall prevalence of SGA in the study population is approximately 20%.

^f^ Includes patent ductus arteriosus (*n* = 65), patent foramen ovale (*n* = 47) and ventricular/ atrial septum defects (*n* = 8); 20 infants had > 1 defect.

^g^ Z-scores calculated according to the WHO Growth Standards, using corrected age.

### Growth Patterns and associations with early-life factors and anthropomorphic outcomes at one year

**Fig. 1:**
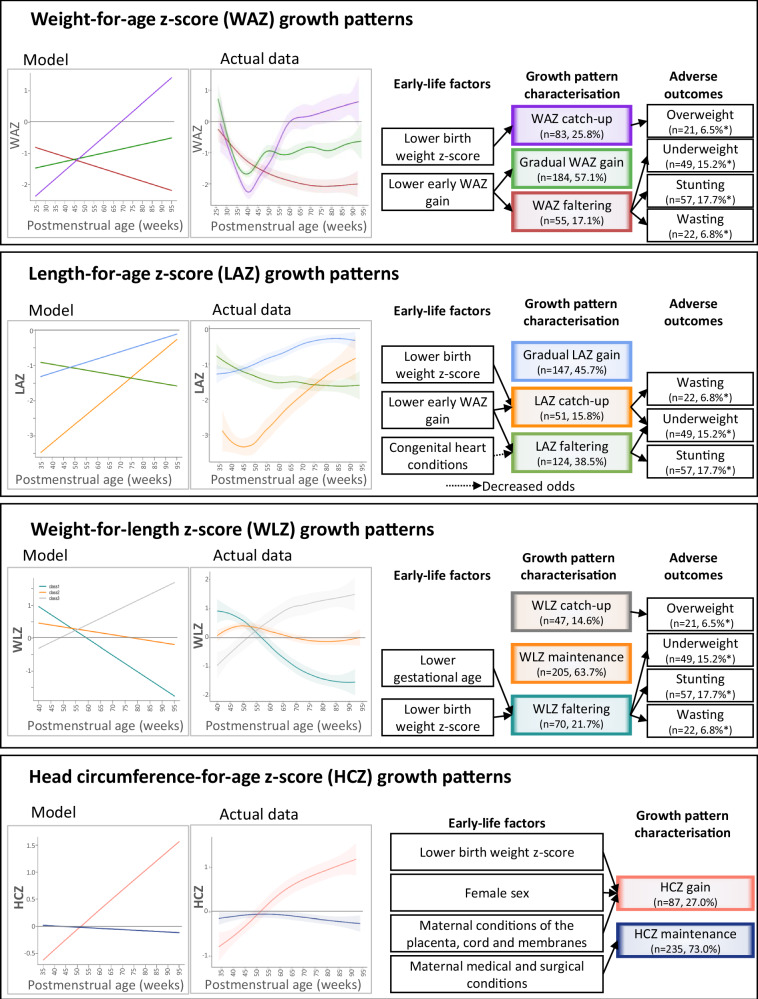
Anthropometric z-score growth patterns (based on Growth Mixture Models and plotted using actual sample data) and corresponding associations with early-life factors and adverse 1-year anthropometric outcomes in 322 preterm infants.

The best models for all indices were achieved using GMM with per-class random intercepts, incorporating a non-linear link function only for HCZ. Figure 1 shows plots of the models’ predicted values and actual data. Table 2 describes infant characteristics per growth pattern group.

**Table 2:** Characteristics of infants with different growth patterns.

Characteristic	Growth pattern characterisations per growth index z-score										
	Weight-for-age (WAZ)			Length-for-age (LAZ)			Weight-for-length (WLZ)			HC-for age (HCZ)	
	Faltering (*n* = 55)	Gradual gain (*n* = 184)	Catch-up (*n* = 83)	Faltering (*n* = 124)	Gradual gain (*n* = 147)	Catch-up (*n* = 51)	Faltering (*n* = 70)	Maintenance (*n* = 205)	Catch-up (*n* = 47)	Maintenance (*n* = 235)	Catch-up (*n* = 87)
*Maternal*											
Maternal age (years) [mean ± SD] (*n* = 285)	30.8 ± 7.2	29.4 ± 6.4	28.8 ± 6.5	29.1 ± 6.6	30.2 ± 6.6	28.3 ± 6.6	30.8 ± 7.2	29.3 ± 6.3	28.3 ± 6.7	29.8 ± 6.6	28.6 ± 6.5
▪ Adolescent ( ≤ 19 years) [*n* (%)]	3/52 (5.8)	8/159 (5.0)	7/74 (9.5)	9/113 (7.9)	6/131 (4.6)	3/41 (7.3)	4 (6.2)	9 (5.1)	5 (11.9)	13/214 (6.1)	5/71 (6.9)
▪ Normal/ low risk (20-34 years) [*n* (%)]	32/52 (61.5)	110/159 (69.2)	51/74 (68.9)	79/113 (69.3)	84/131 (64.6)	30/41 (73.2)	39 (60.0)	123 (69.1)	31 (73.8)	141/214 (66.2)	52/71 (72.2)
▪ Advanced ( ≥ 35 years) [*n* (%)]	17/52 (32.7)	411/59 (25.8)	16/74 (21.6)	26/113 (22.8)	40/131 (30.8)	8/41 (19.5)	22 (33.8)	46 (25.8)	6 (14.3)	59/214 (27.7)	15/71 (20.8)
Parity [median (IQR)] (*n* = 285)	2 (1.75; 3)	2 (1; 3)	2 (1; 3)	2 (1; 3)	2 (1;3)	2 (1; 2)	2 (1;3)	2 (1;3)	2 (1;3)	2 (1; 3)	2 (1; 3)
▪ Primipara, *P* = 1 [*n* (%)]	13/52 (25.0)	58/159 (36.5)	26/74 (35.1)	38/113 (33.6)	43/131 (32.8)	16/41 (39.0)	19 (20.2)	66 (37.1)	12 (228.6)	40/214 (32.7)	27/71 (38.0)
Gravidity [median (IQR)] (*n* = 285)	2 (2; 4)	2 (2; 3)	2 (1; 3)	2 (2; 3)	3 (2; 3)	2 (1; 3)	2 (2;4)	2 (2;3)	2 (1.25;3)	2 (2; 3)	2 (1; 3)
▪ Primigravida, G = 1 [*n* (%)]	9/52 (17.3)	37/159 (23.3)	20/74 (27.0)	25/113 (22.1)	29/131 (22.1)	12/41(29.3)	12 (18.5)	43 (24.2)	11 (26.2)	44/214 (20.6)	22/71 (31.0)
Maternal HIV infection [*n* (%)]	10 (18.2)	42 (22.8)	11 (13.2)	24 (19.4)	30 (20.4)	9 (17.6)	13 (19.1)	44 (23.0)	6 (14.0)	49 (20.9%)	14 (16.1%)
▪ ART in pregnancy	8 (80.0)	29 (69.0)	7 (63.6)	16 (66.7)	20 (66.7)	8 (88.9)	12 (17.6)	28 (14.7)	4 (9.3)	35 (71.4)	9 (64.3)
▪ no ART in pregnancy	2 (20.0)	7 (16.7)	1 (9.1)	7 (29.2)	2 (6.7)	1 (11.1)	0	10 (5.2)	0	8 (16.3)	2 (14.3)
▪ ART timing not recorded	0	6 (14.3)	3 (27.3)	1 (4.2)	8 (26.7)	0	1 (1.5)	6 (3.1)	2 (4.7)	6 (12.2)	3 (21.4)
Maternal conditions ^a^ [*n* (%)]											
▪ … of placenta, cord, membranes	0	9 (4.9)	4 (4.8)	6 (4.8)	5 (3.4)	2 (3.9)	0 (0.0)	9 (4.4)	4 (8.5)	5 (2.1)	8 (9.2)
▪ … of pregnancy	8 (14.5)	37 (20.1)	15 (18.1)	18 (14.5)	32 (21.8)	10 (19.6)	15 (21.4)	36 (17.6)	9 (19.1)	43 (18.3)	17 (19.5)
▪ … of labor and delivery	11 (20.0)	37 (20.1)	16 (19.3)	26 (21.0)	31 (21.1)	7 (13.7)	11 (15.7)	45 (22.0)	8 (17.0)	46 (19.6)	18 (20.7)
▪ Medical and surgical conditions	22 (40.0)	69 (37.5)	23 (27.7)	46 (37.1)	56 (38.1)	12 (23.5)	23 (32.9)	79 (38.5)	12 (25.5)	92 (39.1)	22 (25.3)
*Infant*											
Sex (male) [*n* (%)]	34 (61.8)	87 (47.3)	39 (47.0)	69 (55.6)	69 (46.9)	22 (43.1)	41 (58.6)	99 (48.3)	20 (42.6)	126 (53.6)	34 (39.1)
Gestational age (weeks) [mean ± SD]	32.3 ± 2.8	32.7 ± 2.4	33.2 ± 2.1	33.1 ± 2.4	32.7 ± 2.3	32.2 ± 2.5	32.1 ± 2.6	32.9 ± 2.3	33.2 ± 2.3	32.7 ± 2.5	33.1 ± 2.1
Birth weight z-score ^b^ [mean ± SD]	−0.45 ± 1.01	−0.70 ± 0.90	−1.14 ± 0.94	−0.52 ± 0.90	−0.62 ± 0.77	−1.82 ± 0.92	−1.18 ± 1.00	−0.69 ± 0.96	−0.52 ± 0.71	−0.65 ± 0.92	−1.11 ± 0.97
SGA ^c^ [*n* (%)]	14 (25.5)	52 (28.3)	37 (44.6)	26 (21.0)	38 (25.9)	39 (76.5)	31 (44.3)	64 (31.2)	8 (17.0)	65 (27.7)	38 (43.7)
Infant is one of a set of twins [*n* (%)]	6 (10.9)	33 (17.9)	14 (16.9)	13 (10.5)	30 (20.4)	10 (19.6)	11 (15.7)	34 (16.6)	8 (17.0)	38 (16.2)	15 (17.2)
Any congenital heart condition ^d^ [*n* (%)]	15 (27.3)	58 (31.5)	27 (32.5)	19 (15.3)	52 (35.4)	29 (56.9)	31 (44.3)	57 (27.8)	12 (25.5)	66 (28.1)	34 (39.1)
Early WAZ gain on FGC [mean ± SD]	−0.63 ± 1.01	−0.09 ± 1.04	0.25 ± 1.30	−0.10 ± 0.99	0.12 ± 1.15	−0.72 ± 1.25	−1.18 ± 1.00	−0.69 ± 0.96	−0.52 ± 0.71	−0.08 ± 1.16	−0.14 ± 1.11

*FGC* Fenton growth chart, *HIV* human immunodeficiency virus, *ART* antiretroviral therapy, *SGA* small-for-gestational age, *WAZ* weight-for-age z-score, *LAZ* length-for-age z-score, *WLZ* weight-for-length z-score, *BMIZ* body mass index-for-age z-score

^a^ Maternal conditions classified according to WHO ICD10-PM categories [42]. Conditions of labor and delivery only includes conditions other than preterm delivery, as preterm birth was an inclusion criterion for the study.

^b^ Calculated using the Fenton 2013 Growth Chart [20].

^c^ SGA = small-for-gestational age: birth weight <10^th^ percentile on the Fenton Growth Chart.

^d^ Includes patent ductus arteriosus (*n* = 65), patent foramen ovale (*n* = 47) and ventricular/ atrial septum defects (*n* = 8).

^e^ Anthropometric z-scores calculated according to the WHO Growth Standards, using corrected age

The best-fit WAZ model identified three growth patterns: gradual WAZ gain (*n* = 184, 57.1%), WAZ catch-up (*n* = 83, 25.8%) and WAZ faltering (*n* = 55, 17.1%), shown in Fig. 1. Plots of the actual data showed initial WAZ loss in all growth patterns, followed by partial regain in the gradual WAZ gain pattern and regain to above BWZ in the WAZ catch-up pattern. In regression analysis (Table 3), using WAZ catch-up as the comparator, smaller early WAZ gains (up to 50 weeks PMA) were associated with WAZ faltering (OR 2.27 (1.56–3.33) per 1 less z-score gained) and gradual WAZ gain (OR 1.47 (1.11–1.96) per 1 less z-score gained), while lower BWZ was associated with lower odds of WAZ faltering (OR 0.36 (0.46–0.40) per 1 z-score decrease) and gradual WAZ gain (OR 0.56 (0.53–0.92) per 1 z-score decrease), implying that lower BWZ is associated with increased odds of WAZ catch-up. At 1 year, infants in the WAZ faltering group had the highest rate of underweight (27 underweight infants out of 55 infants with WAZ faltering, 49.1%, compared to 4.8% of the WAZ catch-up group and 9.8% of the gradual WAZ gain group). This was also true for stunting (25 stunted infants out of 55, 45.5%; compared to 4.9% of the WAZ catch-up group and 15.2% of the gradual WAZ gain group) and wasting (12 wasted infants out of 55, 21.8%; compared to 3.7% of the WAZ catch-up group and 3.8% of the gradual WAZ gain group). On the other hand, the WAZ catch-up pattern was associated with proportionally more overweight infants (20 overweight infants out of 83 infants with WAZ catch-up, 24.4%; compared to 1 infant in the gradual WAZ gain group and none in the WAZ faltering group) (All with *p* < 0.001) (Table 4).

**Table 3:** Association of prenatal, perinatal and early-life predictors with growth patterns: odds ratios calculated by univariate and multivariate analysis.

WEIGHT-FOR-AGE Z-SCORE (WAZ)					
Baseline comparator: WAZ catch-up pattern	Odds (95%CI) of WAZ faltering pattern		Odds (95%CI) of gradual WAZ gain pattern		Model R^2^
Predictors:	Univariate analysis	Multivariate model	Univariate analysis	Multivariate model	
▪ Lower gestational age at birth (per 1 week decrease)	1.19 (1.03, 1.37) *	NS	NS	NS	0.072
▪ Lower birth weight z-score ^a^ (per 1 z-score decrease)	0.45 (0.43. 0.40) ***	0.36 (0.19, 0.68) **	0.61 (0.83, 0.81) ***	0.56 (0.53, 0.92) *	
▪ SGA ^b^	0.42 (0.20, 0.89) *	NS	0.49 (0.29, 0.84) **	NS	
▪ Lower early WZ gain ^c^ (per 1 z-score decrease)	2.04 (1.47, 2.86) ***	2.27 (1.56, 3.33) ***	1.33 (1.05, 1.72) *	1.47 (1.11, 1.96) **	
**LENGTH-FOR-AGE Z-SCORE (LAZ)**					
**Baseline comparator: Gradual LAZ gain pattern**	**Odds (95%CI) of LAZ faltering pattern**		**Odds (95%CI) of LAZ catch-up pattern**		**Model R**^**2**^
**Predictors:**	**Univariate analysis**	**Multivariate model**	**Univariate analysis**	**Multivariate model**	
▪ Lower birth weight z-score ^a^ (per 1 z-score decrease)	NS	NS	6.25 (3.45, 11.11) ***	8.33 (3.13, 20.00) ***	0.193
▪ SGA ^b^	NS	NS	9.32 (4.42, 19.63) ***	NS	
▪ Infant is one of a set of twins	0.46 (0.23, 0.92) *	NS	NS	NS	
▪ Any congenital heart condition	0.33 (0.18, 0.60) ***	0.28 (0.14, 0.53) ***	2.41 (1.26, 4.61) **	NS	
▪ Lower early WZ gain ^c^ (per 1 z-score decrease)	NS	1.39 (1.09, 1.75) ***	1.96 (1.45, 2.63) ***	1.85 (1.25, 2.70) **	
**WEIGHT-FOR-LENGTH Z-SCORE (WLZ)**					
**Baseline comparator: WLZ maintenance pattern**	**Odds (95%CI) of WLZ faltering pattern**		**Odds (95%CI) of WLZ gain pattern**		**Model R**^**2**^
**Predictors:**	**Univariate analysis**		**Multivariate model**		
▪ Any congenital heart condition	2.06 (1.18, 3.62)*	NS	NS	NS	0.070
▪ Lower gestational age at birth (per 1 week decrease)	1.13 (1.02, 1.27)*	1.26 (1.10, 1.45)**	NS	NS	
▪ Lower birth weight z-score ^a^ (per 1 z-score decrease)	1.75 (1.11, 3.33)***	2.94 (1.69, 5.00)***	NS	NS	
▪ SGA ^b^	1.75 (1.00, 3.06)*	NS	NS	NS	
**HEAD CIRCUMFERENCE-FOR-AGE Z-SCORE (HCZ)**					
**Baseline comparator: HCZ maintenance pattern**	**Odds (95%CI) of HCZ catch-up pattern**				**Model R**^**2**^
**Predictors:**	**Univariate analysis**		**Multivariate model**		
▪ Maternal conditions of placenta, cord, membranes ^d^	4.66 (1.51, 15.81) **		5.70 (1.80, 19.87) ***		0.095
▪ Maternal medical and surgical conditions ^d^	0.53 (0.30, 0.90) *		0.49 (0.27, 0.86) *		
▪ Female sex	1.79 (1.10, 3.03) *		1.85 (1.09, 3.13) *		
▪ Lower birth weight z-score ^a^ (per 1 z-score decrease)	1.69 (1.30, 2.27) ***		1.92 (1.23, 3.13) **		
▪ SGA ^b^	1.99 (1.19, 3.32) **		NS		

NS: not significant. Odds ratios are only shown for variables with significant associations.

Variables included in all the analyses: Maternal age; maternal age category (adolescent ≤ 19 years, low risk 20–34 years, advanced ≥ 35 years); parity; gravidity; maternal HIV infection; timing of ART initiation for mothers with HIV (ART during pregnancy vs. no ART during pregnancy); maternal conditions of the placenta, cord and membranes; maternal conditions of pregnancy; maternal conditions of labor and delivery (other than preterm delivery); maternal medical and surgical conditions, infant sex; gestational age at birth; birth weight z-score; SGA, birth weight <10^th^ percentile; vs. not SGA, birth weight ≥10^th^ percentile); infant is one of a set of twins; infant has a congenital heart condition; early WZ gain (change in weight-for-GA z-score from birth to the last measurement before 50 weeks PMA, calculated using the Fenton 2013 growth chart)

* *p* < 0.05; ** *p* < 0.01; *** *p* < 0.001.

^a^ Calculated using the Fenton 2013 Growth Chart [20].

^b^ SGA = small-for-gestational age: birth weight <10^th^ percentile on the Fenton Growth Chart.

^c^ Change in weight z-score from birth to ≤ 50 weeks PMA, using the Fenton growth chart.

^d^ Maternal conditions classified according to WHO ICD10-PM categories [42]. Only conditions of labour other than preterm delivery were counted, as preterm birth was an inclusion criterion.

**Table 4:** Association between different growth patterns and anthropometric indicators of malnutrition at 1 year (*N* = 322).

Growth pattern	Indicators of malnutrition			
	Underweight (WAZ ^a^ <-2) *N* = 49 *n* (%) ^b^	Stunting (LAZ ^a^ <-2) *N* = 57 *n* (%) ^b^	Wasting (WLZ ^a^ <-2) *N* = 22 *n* (%) ^b^	Overweight (BMIZ ^a^ >+2) *N* = 21 *n* (%) ^b^
WEIGHT-FOR-AGE (WAZ)				
▪ WAZ catch-up *(N* = *83)*	4 (4.8)	4 (4.9)	3 (3.7)	20 (24.4)
▪ Gradual WAZ gain *(N* = *184)*	18 (9.8)	28 (15.2)	7 (3.8)	1 (0.5)
▪ WAZ faltering *(N* = *55)*	27 (49.1)	25 (45.5)	12 (21.8)	0
*p*-value	<0.001 ^c^	<0.001 ^c^	<0.001 ^c^	<0.001 ^c^
**LENGTH-FOR-AGE (LAZ)**				
▪ LAZ catch-up *(N* = *51)*	15 (29.4)	12 (23.5)	9 (17.6)	2 (3.9)
▪ Gradual LAZ gain *(N* = *147)*	3 (2.0)	3 (2.0)	3 (2.0)	14 (9.6)
▪ LAZ faltering *(N* = *124)*	31 (25.0)	42 (33.9)	10 (8.1)	5 (4.0)
*p* value	<0.001 ^c^	<0.001 ^c^	<0.001 ^c^	0.1616 ^c^
**WEIGHT-FOR-LENGTH (WLZ)**				
▪ WLZ faltering *(N* = *70)*	36 (51.4)	26 (37.1)	21 (30.0)	0
▪ WLZ maintenance *(N* = *205)*	12 (5.8)	29 (14.1)	1 (0.5)	6 (2.9)
▪ WLZ catch-up *(N* = *47)*	1 (2.1)	2 (4.3)	0	15 (31.9)
*p*-value	<0.001 ^c^	<0.001 ^c^	<0.001 ^c^	<0.001 ^c^
HC-FOR-AGE (HCZ)				
▪ HCZ maintenance *(N* = *235)*	40 (17.0)	45 (19.2)	19 (8.1)	8 (3.4)
▪ HCZ catch-up *(N* = *87)*	9 (10.3)	12 (13.8)	3 (3.4)	13 (14.9)
*p* value	0.139 ^d^	0.257 ^d^	0.214 ^c^	0.237 ^d^

*BMIZ* body mass index (BMI)-for-age z-score, *HC* head circumference, *HCZ* HC-for-age z-score, *LAZ* length-for-age z-score, *WAZ* weight-for-age z-score, *WLZ* weight-for-length z-score.

^a^ Z-scores calculated according to the WHO Growth Standards, using corrected age.

^b^ Percentages calculated using number of infants in the trajectory group (row N) as denominator.

^c^ Fisher’s Exact Test.

^d^ Chi Squared Test.

The best-fitting LAZ model described three growth patterns: LAZ catch-up (*n* = 51, 15.8%), gradual LAZ gain (*n* = 147, 45.6%) and LAZ faltering (*n* = 124, 38.5%), also illustrated in Fig. 1. All the mean LAZ curves remained below zero, though the LAZ catch-up growth pattern maintained an upward slope at 1 year. In regression analysis using gradual LAZ gain as the baseline comparator (Table 3), lower BWZ was associated with LAZ catch-up (OR 8.33 (3.13–20.00) per 1 z-score decrease), while smaller early WAZ gain (up to 50 weeks PMA) was associated with both LAZ catch-up (OR 1.85 (1.25–2.70) per 1 less z-score gained), and LAZ faltering (OR 1.39 (1.09–1.75) per 1 less z-score gained). Congenital heart conditions were associated with lower odds of LAZ faltering (OR 0.28 (0.14–0.53)). Infants in the gradual LAZ gain group had the lowest rates of all indicators of undernutrition. Only 3 infants out of 147 infants with gradual LAZ gain were underweight (2.0%; compared to 29.4% of infants with LAZ catch-up and 25.0% of infants with LAZ faltering). The same was true for stunting (3 stunted infants out of 147, 2.1%; compared to 23.5% of infants with LAZ catch-up and 33.9% of infants with LAZ faltering) and wasting (3 wasted infants out of 147, 2.1%; compared to 17.6% of infants with LAZ catch-up and 8.1% of infants with LAZ faltering) at 1 year (Table 4). LAZ catch-up was associated with the highest rates of underweight (29.4%) and wasting (17.6%), and LAZ faltering with the largest percentage of stunting (33.9%; all *p* < 0.001). The rates of overweight did not differ significantly between the different LAZ patterns.

The best-fitting WLZ model described two growth patterns, namely WLZ gain and WLZ faltering. However, the next-best fitting model was considered more appropriate to what is observed in the clinical setting, as it described three patterns: WLZ faltering (*n* = 70, 21.7%), WLZ maintenance (*n* = 205, 63.7%) and WLZ catch-up (*n* = 47, 14.6%), as seen in Fig. 1. The WLZ faltering curve declined consistently from ~50 weeks PMA ( ~ 10 weeks CA), and flattening from ~80 weeks PMA (9 months CA). In multiple regression analysis, the odds of WLZ faltering were significantly increased by lower birth GA (OR 1.26 (1.10–1.45) per 1 week decrease) and lower BWZ (OR 2.94 (1.69–5.00) per 1 z-score decrease). Unsurprisingly, WLZ faltering was associated with indicators of undernutrition, with all but one of the wasted infants displaying WLZ faltering (21 wasted infants out of 70 (30.0%); compared to one infant (0.5%) with WLZ maintenance and zero wasted infants in the WLZ catch-up group). WLZ faltering was likewise associated with underweight (36 underweight infants out of 70 infants with WLZ faltering, 54.1%; compared to 12 infants (5.8%) with WLZ maintenance and one infant (2.1%) with WLZ catch-up) and stunting (26 stunted infants out of 70, 37.1%; compared to 14.1% of infants with WLZ maintenance and 4.3% of infants with WLZ catch-up). Conversely, WLZ catch-up was associated with overweight (15 overweight infants out of 47 infants with WLZ catch-up, 31.9%; compared to 2.9% of infants with WLZ maintenance and none of the infants with WLZ faltering) (All *p* < 0.001) (Table 4).

The best-fitting HCZ model described two growth patterns: HCZ maintenance near zero (*n* = 87, 27.0%), and HCZ catch-up (*n* = 235, 73.0%) to HCZ > 1 at 1 year, illustrated in Fig. 1. The odds of HCZ catch-up increased with lower BWZ (OR 1.92 (1.23–3.13) per 1 z-score decrease), female sex (OR 1.85 (1.09–3.13)) and maternal conditions of the placenta, cord, and membranes (OR 5.70 (1.80–19.87)), but decreased with maternal medical and surgical conditions (OR 0.49 (0.27–0.86)) (Table 3). Neither HCZ growth pattern was significantly associated with indicators of malnutrition (Table 4).

## Discussion

This study illustrates a variety of postnatal growth patterns for preterm infants in a LMIC setting who received KMC in early life. Certain birth and early life factors were associated with different growth patterns, and certain growth patterns may be useful for identifying infants at risk of underweight, stunting, wasting and overweight at 1 year. This could have clinical utility for flagging infants at risk of adverse growth outcomes, facilitating timely support and appropriate intervention.

For each anthropometric indicator, over 80% of the infants fell within the healthy age-corrected z-score range of -2 to +2 at 1 year of age, where one would expect ~95% of the children in an optimally nourished population to fall. The relatively high rates of excess underweight, stunting and wasting (above the expected ~2.3%) in the sample are not unexpected, given the well-described risks of sub optimal growth outcomes in SGA and preterm infants [1–3]. Furthermore, catch-up growth is likely to be incomplete at 1 year of age, and can reasonably be expected to continue through the second year of life; thus, the rates of underweight and stunting may well decrease further over time. In a large Canadian study that followed early preterm infants (GA 24–29 weeks) up to 3 years CA, the numbers of infants with WAZ or LAZ < -2 decreased progressively from 4 to 36 months CA [21], although the study included a much smaller proportion of SGA infants (4.9%) than our sample (32.0%). The substantial catch-up growth in our study sample is further illustrated by the fact that the rate of stunting, at 17.0%, is substantially lower than the reported South African population prevalence of stunting (31.4% in infants 12–17 months old) [22]. Taken together, this provides support for early, prolonged KMC, strong breastfeeding support and regular infant follow-up as strategies to optimise preterm infant growth in a resource-constrained LMIC setting.

Over 80% of infants were classified as having acceptable WAZ growth patterns (i.e. gradual WAZ gain (184/322, 57.1%) or WAZ catch-up (83/322, 25.8%)). While the gradual WAZ gain pattern did not reach BWZ, the plateau level was above the lowest WAZ, possibly reflecting physiologic postnatal weight loss and regain. Consistent with research showing that few preterm infants regain their BWZ by 40 weeks PMA [23–26], the gradual WAZ gain and WAZ catch-up growth patterns only started increasing after term age. Guidelines for preterm infant growth targets recommend at least maintaining the WAZ at which weight gain starts, even if the BWZ is not regained [23–26]. More than 60% of the infants had appropriate LAZ growth (i.e. gradual LAZ gain (147/322, 45.7%) or LAZ catch-up (51/322, 15.8%)), yet a considerable number exhibited LAZ faltering (124/322, 38.5%). Preterm infants with insufficient LAZ catch-up growth are likely to remain stunted throughout childhood. With an estimated preterm birth rate of 13% ( ~ 150,000 preterm births annually) [27], LAZ faltering in preterm infants is of particular concern as a possible contributor to childhood stunting, which remains prevalent in South Africa [22]. For WLZ, the majority of the infants displayed a WLZ maintenance pattern (205/322, 63.7%), with a further 47/322 (14.6%) attaining WLZ catch-up, which may be considered a more appropriate growth pattern in this sample due to the large proportion of SGA neonates. The 47 infants (14.6%) who displayed WLZ faltering are a group of concern, particularly given the strong association between WLZ faltering and wasting at 1 year in this sample. Though most infants exhibited acceptable growth and anthropometric status at 1 year, it is important for the clinician to be cognisant of growth patterns that may indicate an increased risk of malnutrition. In this regard, infants with WAZ, LAZ and WLZ faltering represent the groups of highest concern for undernutrition at 1 year (Table 4). Identifying the early-life factors that are associated with these growth patterns may therefore be useful in flagging infants whose growth should be monitored more closely.

Of the early-life predictors, BWZ and early WAZ gain (up to 50 weeks PMA) were most consistently associated with 1-year growth patterns. Lower BWZ was associated with WAZ and LAZ catch-up, consistent with literature describing higher postnatal growth velocity in SGA infants compared to AGA infants [7, 28, 29]. In this sample, SGA infants had considerably smaller WAZ gains up to 50 weeks PMA (−0.36 ± 1.22 vs. 0.04 ± 1.08 z-scores, Supplementary Table 1), in contrast to other research showing greater early growth in SGA infants [29]. However, SGA infants did have greater overall WAZ gain in the first year of life (0.47 ± 1.20 vs. 0.08 ± 1.31 z-scores, Supplementary Table 1). In the context of lower BWZ following intrauterine growth faltering due to placental insufficiency [30–33], adequate postnatal nutrition can result in accelerated growth and return to the genetic growth potential [23]. Conversely, lower BWZ was also associated with WLZ faltering, raising the possibility that WLZ faltering may co-exist with seemingly adequate WAZ and/ or LAZ growth. Literature has long described sub-optimal long-term growth outcomes in SGA infants [1–3, 5, 34–36]. In univariate analyses, SGA performed similarly to BWZ, but associations became non-significant when SGA and BWZ were both included in multiple regression analysis. This may imply that BWZ predicts growth patterns even in non-SGA infants, particularly since WAZ and LAZ faltering were associated with higher rather than lower BWZ. Our results furthermore suggest that, despite the more rapid postnatal WAZ and LAZ growth experienced by smaller infants, catch-up growth was still incomplete at 1 year, and disproportionate WAZ and LAZ growth may still result in WLZ faltering. This further emphasises the importance of longitudinal WLZ growth monitoring, and argues against SGA as an isolated indicator of malnutrition risk.

Smaller early WAZ gains (up to 50 weeks PMA) were associated with WAZ faltering, slow WAZ growth, LAZ faltering and LAZ catch-up, while the greatest early WAZ growth was associated with WAZ catch-up and gradual LAZ gain. The associations between early WAZ growth and WAZ patterns are not surprising since one is a subset of the other. The relationship between WAZ and LAZ growth patterns is less well established, though one Bangladeshi study found that stunting at 12 and 24 months was predicted by earlier episodes of weight faltering [37]. The WAZ and LAZ catch-up growth patterns were associated in the lowest rates of underweight, wasting and stunting, but the highest rates of overweight. This has been observed in previous studies, where early growth was protective against undernutrition, but excessive early weight gain was associated with later overweight [38]. Research in various LMICs likewise identified failure to regain birth weight by two weeks of age (indicating insufficient early growth) as a risk factor for stunting and underweight at 6 months old in term and preterm low birth weight infants [34].

Lower birth GA was associated with WLZ faltering, which was in turn associated with higher rates of underweight, stunting and wasting. Preterm birth is a well-established risk factor for long-term growth deficits, particularly when complicated by SGA [1–4, 6].

Infant congenital heart conditions were only associated with LAZ growth patterns, with an increased odds of LAZ catch-up and decreased odds of LAZ faltering (compared to gradual LAZ gain). Though congenital heart conditions were also associated with WLZ faltering in univariate analysis, the association became non-significant in multivariable analysis, suggesting that this association may be mediated by lower GA or lower BWZ (both of which were associated with congenital heart conditions in this sample). Congenital heart conditions are more often associated with sub-optimal growth outcomes, and in this sample the infants with congenital heart conditions were smaller at 1 year. However, they also had lower birth weight z-scores and gestational age at birth, which emerged as significant predictors of WAZ and WLZ growth patterns in multivariate analysis, while congenital heart conditions did not. This may be attributed to the fact that the congenital heart conditions observed in the sample were mostly associated with delayed adaptation to preterm birth (PDA, 65/100 cases and patent foramen ovale PFO, 47/100 cases), and in all but one case resolved early in the neonatal course, either spontaneously or with the administration of non-steroidal anti-inflammatory drugs. Minor cardiac defects like PDA and PFO are often associated with preterm birth; thus, it is reassuring to know that in this sample of infants they were not associated with sub-optimal postnatal growth patterns. Ventricular/ atrial septum defects (present in eight infants in this sample) are potentially more problematic, and deserve investigation in a larger sample.

The lack of a distinct HCZ faltering growth pattern is reassuring, as HC growth predicts neurodevelopmental outcomes in preterm infants [39, 40]. Our research did not assess neurodevelopment, but HCZ growth pattern was not associated with 1-year malnutrition.

To the best of our knowledge, this is the first study to use LCTM to characterise longitudinal WAZ, LAZ, WLZ and HCZ growth patterns in South African preterm infants. This research is potentially useful in clinical practice, facilitating the identification of particularly nutritionally at-risk preterm infants by characterising early-life risk factors and growth patterns associated with adverse growth outcomes. However, the study sample cannot be considered representative of all South African preterm infants, as it was limited to a small geographic area, and included a larger-than-typical proportion of SGA infants. The sample size was also insufficient to allow for detailed investigation of the association of infant comorbidities with growth patterns, particularly for less prevalent conditions. Moreover, routine clinical data has some inherent limitations, particularly regarding the completeness of maternal health information. The lack of reliable birth length and HC measurements is another important limitation that should be addressed in future research and clinical practice. Finally, while not all infants require catch-up growth, for those who do, 1 year was likely insufficient to capture the full extent of catch-up growth, which may be expected to continue up to 2 years of age. This is particularly apparent in the LAZ catch-up growth pattern, which still had a steep upward slope by the end of the study period. Likewise, capturing the development of overweight/obesity in this population will require longer follow-up.

Many questions remain for future research. At the most basic level, a clear definition of appropriate early catch-up growth remains elusive, as any definition of catch-up growth would have to consider the selection of growth indices to consider, the magnitude of the z-score gain, and the time over which the growth took place. Due to the computational complexity, machine learning and automated pattern recognition methods may be useful to assist in delineating the limits of appropriate growth versus growth faltering in relation to key outcomes. The role of infant feeding practices and dietary intake deserve special attention, not only as predictors of growth but also as potential interventions to promote more favourable growth trajectories. Body composition assessment would also be valuable, since it is known that preterm infants tend to have lower fat-free mass and higher fat mass percentage than term-infants at term-equivalent age [25]. Knowing how body composition evolves throughout childhood and beyond would offer important insights into the metabolic risks associated with different growth patterns.

## Conclusion

Preterm infants display a variety of growth patterns, including catch-up growth, gradual growth, and growth faltering. Most of the infants studied had appropriate growth and attained normal anthropometric status at 1 year of age, highlighting that KMC, breastfeeding support and regular postnatal follow-up can support desirable growth. Growth faltering and the concomitant risk of stunting and wasting remain a concern in a subset of infants. The prediction of poor growth outcomes in preterm infants is complex; early life factors and growth patterns offer important insights, yet growth should always be considered with the overall clinical picture.

Lower BWZ was associated with WAZ and LAZ catch-up in the first year of life, but also with WLZ faltering, highlighting the importance of monitoring all three growth indices concurrently. While WAZ catch-up was associated with overweight at 1 year, LAZ catch-up and WLZ faltering were more strongly associated with underweight and wasting. Smaller early WAZ gains (up to 50 weeks PMA) were associated with WAZ and LAZ faltering, which were in turn associated with underweight, stunting and wasting. Conversely, gradual WAZ and LAZ gain were associated with low rates of malnutrition at 1 year, suggesting that these are appropriate growth trajectories in preterm infants. Though WAZ catch-up was associated with even lower rates of malnutrition than gradual WAZ gain, it was also associated with overweight, indicating the importance of monitoring WLZ alongside WAZ.

Longitudinal, comprehensive monitoring of preterm infants’ growth trajectories presents opportunities for timely interventions to promote their growth and development.

## Supplementary information

Supplementary table

## Data Availability

Data available from authors upon reasonable request.
