# Supplementary material for: First-year growth patterns of preterm infants receiving kangaroo mother care: associations with early life factors and 1-year anthropometry
Source: Eur J Clin Nutr. 2025 Oct 15;80(1):62–72. doi: 10.1038/s41430-025-01662-6 (PMC12783048; doi:10.1038/s41430-025-01662-6)
Supplement: Supplementary file 1 — Supplementary table [file 41430_2025_1662_MOESM1_ESM.docx]

# Supplementary Table: Comparison maternal and infant characteristics at birth and one year of SGA, AGA and LGA infants

| Characteristic |  | Value | | |
| --- | --- | --- | --- | --- |
|  | **N** | **SGA (n=103)** | **AGA (n=216)** | **LGA (n=3)** |
| ***Maternal characteristics ^a^*** | | | | |
| Maternal age (years) [Mean ± SD] | 287 | 29.3 ± 6.6 | 29.5 ± 6.6 | 32.7 ± 1.5 |
| - Adolescent: age ≤19 years [n (%)] |  | 6/86 (7.0) | 13/196 (6.6) | 0 |
| - Advanced maternal age: ≥35 years [n (%)] |  | 20/86 (23.3) | 54/196 (27.3) | 0 |
| Gravidity (number of pregnancies) [median (IQR)] | 287 | 2 (1, 3) | 2 (1, 3) | 3 (2.5, 3.5) |
| - Primigravida, Gravidity=1 [n (%)] |  | 25/86 (29.1) | 42/198 (21.2) | 1/3 (333) |
| Parity (number of pregnancies carried to viable gestational age) [median (IQR)] | 287 | 2 (1, 3) | 2 (1, 3) | 3 (2.5, 3.5) |
| - Primipara, Parity=1 [n (%)] |  | 33/86 (38.4) | 63/198 (31.8) | 1/3 (33.3) |
| Maternal HIV infection [n (%)] | 302 | 27/100 (27.0) | 36/199 (18.1) | 0 |
| - Received ART during pregnancy |  | 17/27 (63.0) | 27/36 (75.0) | 0 |
| - No ART during pregnancy |  | 6/27 (22.2) | 4/36 (11.1) | 0 |
| - ART not recorded |  | 4/27 (14.8) | 5/36 (13.9) | 0 |
| Maternal conditions during pregnancy ^b^ [n (%)] | 302 |  |  |  |
| - Conditions of the placenta, cord, membranes |  | 3/100 (3.0) | 10/199 (5.0) | 0 |
| - Pregnancy conditions |  | 22/100 (22.0) | 39/199 (19.6) | 0 |
| - Labor and delivery conditions |  | 16/100 (16.0) | 46/199 (23.1) | 1/3 (33.3) |
| - Medical and surgical conditions |  | 43/100 (43.0) | 69/199 (34.7) | 2/3 (66.7) |
| ***Infant characteristics at birth*** | | | | |
| Infant sex (male) [n (%)] | 322 | 53 (51.5) | 105 (48.6) | 2 (66.7) |
| Gestational age (weeks) [Mean ± SD] | 322 | 33.5 ± 2.2 | 32.5 ± 2.4 | 32.3 ± 3.2 |
| Birth weight (kg) [Mean ± SD] | 322 | 1.39 ± 0.42 | 0.20 ± 1.24 | 2.72 ± 1.29 |
| - Birth weight z-score ^c^ [Mean ± SD] |  | -1.82 ± 0.60 | -0.31 ± 0.57 | 2.29 ± 0.86 |
| Infant is one of a set of twins [n (%)] | 322 | 20 (19.4) | 33 (15.3) | 0 |
| Infant congenital heart conditions ^d^ [n (%)] | 322 | 42 (40.8) | 57 (26.4) | 1 (33.3) |
| ***Infant characteristics at one year*** | | | | |
| Chronological age (months) [Mean ± SD] | 322 | 12.5 ± 0.6 | 12.6 ± 0.6 | 12.7 ± 0.7 |
| Corrected age (months) [Mean ± SD] | 322 | 11.0 ± 0.7 | 10.9 ± 0.8 | 11.0 ± 0.4 |
| Still breastfeeding at last visit [n (%)] | 322 | 61/103 (59.2) | 120/216 (22.6) | 3/3 (100.0) |
| Change in WAZ from birth to ≤ 50 weeks PMA^c^ [Mean ± SD] | 320 | -0.36 ± 1.22 | 0.04 ± 1.08 | -1.19 ± 0.70 |
| Weight (kg) [Mean ± SD] | 322 | 7.86 ± 1.22 | 8.92 ± 1.40 | 9.37 ± 1.28 |
| - Weight-for-age z-score ^e^ [Mean ± SD] | 322 | -1.33 ± 1.23 | -0.21 ± 1.28 | 0.18 ± 0.90 |
| - Weight-for-length z-score ^e^ [Mean ± SD] | 321 | -0.74 ± 1.23 | 0.15 ± 1.26 | 0.10 ± 0.89 |
| - BMI-for-age z-score ^e^ [Mean ± SD] | 321 | -0.62 ± 1.24 | 0.20 ± 1.24 | 0.08 ± 0.85 |
| Length (cm) [Mean ± SD] | 321 | 69.9 ± 2.9 | 72.0 ± 2.8 | 74.3 ± 2.1 |
| - Length-for-age z-score ^e^ [Mean ± SD] | 321 | -1.53 ± 1.04 | -0.61 ± 1.11 | 0.20 ± 0.55 |
| Head circumference (cm) [Mean ± SD] | 322 | 44.9 ± 1.9 | 45.6 ± 1.7 | 45.8 ± 2.2 |
| - HC-for-age z-score ^e^ [Mean ± SD] | 322 | -0.20 ± 1.43 | 0.41 ± 1.16 | 0.35 ± 1.22 |
| Indicators of malnutrition [n (%)] |  |  |  |  |
| - Underweight: Weight-for-age z-score ^e^ <‑2 | 322 | 34/103 (33.0) | 15/216 (6.9) | 0 |
| - Stunted: Length-for-age z-score ^e^ <‑2 | 321 | 33/103 (32.0) | 24/216 (11.1) | 0 |
| - Wasted: Weight-for-length z-score ^e^ <‑2 | 321 | 13/103 (12.6) | 9/216 (4.2) | 0 |
| - Overweight: BMI-for-age z-score ^e^ >+2 | 321 | 3/103 (2.9) | 18/216 (8.3) | 0 |
| ^a^ 20 duplicate records of mothers of 40 twins were removed: thus N=302 mothers.  ^b^ Maternal conditions classified according to WHO ICD10-PM categories^1^. Conditions of labor and delivery only includes conditions other than preterm delivery, as preterm birth was an inclusion criterion for the study.  ^c^ Calculated using the Fenton 2013 Growth Chart^2^  ^d^ Includes patent ductus arteriosus (n=65), patent foramen ovale (n=47) and ventricular/ atrial septum defects (n=8); 20 infants had >1 defect.  ^e^ Z-scores calculated according to the WHO Growth Standards, using corrected age.  Abbreviations: AGA = appropriate-for-gestational age; ART = antiretroviral therapy; BMI = body mass index, HC = head circumference; HIV = human immunodeficiency virus; LGA = large-for-gestational age SGA = small-for-gestational age; WAZ = weight-for-age z-score. | | | | |

References:

1. World Health Organization. The WHO application of ICD-10 to deaths during the perinatal period: ICD-PM. Geneva: World Health Organization; 2016. Available from: <https://www.who.int/publications/i/item/9789241549752>.

2. Fenton TR, Kim JH. A systematic review and meta-analysis to revise the Fenton growth chart for preterm infants. BMC Pediatr. 2013; 13:59-2431-13-59. doi: 10.1186/1471-2431-13-59.
